# Supplementary figures and images for: Monosynaptic Projections to Excitatory and Inhibitory preBötzinger Complex Neurons
Source: Front Neuroanat. 2020 Sep 4;14:58. doi: 10.3389/fnana.2020.00058 (PMC7507425; doi:10.3389/fnana.2020.00058)

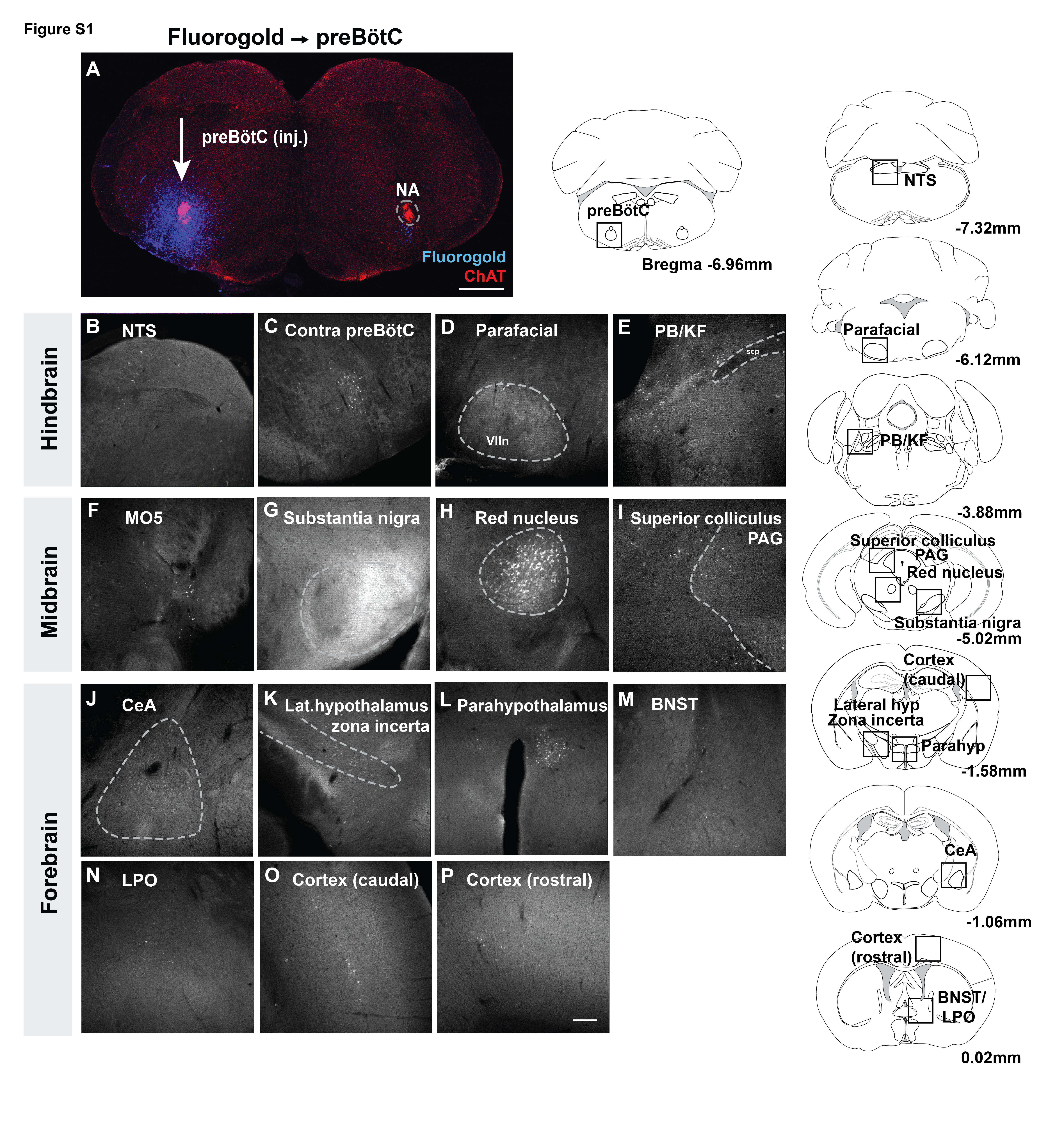

Supplement: FIGURE S1 — Retrograde labeling of putative afferent projections to the preBötC. Fluorogold injections into the preBötC [(A), arrow] result in retrograde labeling of neurons throughout the brain including the nucleus of the solitary tract (NTS) contralateral preBötC, parafacial and facial (VIIn) nuclei, parabrachial nuclei and Kölliker-Fuse (PB/KF), trigeminal nucleus (MO5), substantia nigra, red nucleus, superior colliculus, central amygdala, lateral hypothalamus, and zona incerta, paraventricular hypothalamus, bed nucleus of the stria terminalis (BNST), lateral preoptic area (LPO), and cortex (B–P). (ChAT staining, red), (scp, superior cerebellar peduncle) (A) Scale bar = 500 μm; (B–P) Scale bar = 200 μm. [file Image_1.JPEG]
